# Development of an appropriate simple suspension method for valganciclovir medication

**DOI:** 10.1186/s40780-020-00172-w

**Published:** 2020-07-07

**Authors:** Yasuyuki Masaoka, Yoichi Kawasaki, Ryo Kikuoka, Atsushi Ogawa, Satoru Esumi, Yudai Wada, Soichiro Ushio, Yoshihisa Kitamura, Toshiaki Sendo

**Affiliations:** 1 Department of Pharmacy, Okayama University Hospital, 2-5-1, Shikata-cho, Kita-ku, Okayama, 700-8558 Japan; 2 Department of Clinical Pharmacy, Okayama University Graduate School of Medicine, Dentistry, and Pharmaceutical Sciences, 2-5-1; Shikata-cho; Kita-ku, Okayama, 700-8558 Japan; 3 Department of Pharmacotherapy, School of Pharmacy, Shujitsu University, 1-6-1, Nishigawara, Naka-ku, Okayama, 703-8516 Japan

**Keywords:** Valganciclovir, Simple suspension method, Stability, HPLC, Gavage tube

## Abstract

**Background:**

Valganciclovir (VGC) is essential for preventing cytomegalovirus infections after transplants in adult and pediatric patients. In pediatric patients, VGC tablets have to be pulverized so that they can be delivered via nasogastric tubes. The “simple suspension method” is usually used to suspend tablets in hot water in Japan. However, the optimal suspension conditions and metering methods for preparing VGC suspensions using the simple suspension method are unclear. The purpose of this study was to clarify these issues.

**Methods:**

VGC tablets were suspended in water (initial water temperature: 25 °C or 55 °C) using the simple suspension method. The residual rate of VGC after it had been suspended in hot water was determined using HPLC. In addition, the suspended solution was passed through 6, 8, and 12 Fr. gavage tubes. The VGC concentrations of suspensions produced using different preparation methods were also determined using HPLC.

**Results:**

Cracking the surfaces of VGC tablets and suspending them in water at an initial temperature of 55 °C was effective at dissolving the tablets. The VGC concentration of the suspension remained stable for at least 80 min. Furthermore, the VGC concentration remained stable for 48 h during cold dark storage. Cracking the surfaces of VGC tablets could be a more effective metering method than preparing powder from VGC tablets. In addition, little VGC remained in 6, 8, or 12 Fr. gavage tubes after VGC solution was passed through them.

**Conclusion:**

The amount of VGC should be measured carefully when preparing VGC solutions using the simple suspension method.

## Background

Cytomegalovirus (CMV) infections are a major cause of morbidity and mortality among post-transplant patients [1, 2]. Early CMV infections are associated with an increased risk of acute graft rejection [3]. Some antiviral drugs can improve graft survival and the outcomes of transplant patients. The antiviral drugs valganciclovir (VGC), acyclovir, valaciclovir, and ganciclovir can be used as prophylaxes against CMV infections. In particular, VGC is the gold-standard treatment for preventing and treating CMV infections in adult and pediatric patients [4, 5].

Medication dosages often have to be adjusted for pediatric patients. The dose (mg) of VGC given to children is calculated as follows: 7 × body surface area (m^2^) × estimated creatinine clearance (mL/min/1.73 m^2^) [4]. VGC is available in tablet form (450-mg VALIXA® tablets) and as a dry syrup preparation (VALIXA® 5000-mg dry syrup) in Japan. Since VGC is used to treat transplant patients, many pharmacies only stock the tablet form. Thus, during the treatment of pediatric patients VGC tablets often have to be pulverized, before being administered via nasogastric tubes (6, 8, or 12 Fr.). Our preliminary research revealed that 9 out of 129 patients (7.0%) that were treated with VGC at Okayama University Hospital in the last year were prescribed pulverized VGC powder (produced by crushing VGC tablets). In particular, pulverized VGC tablets account for approximately 81% of all VGC prescriptions to pediatric patients (< 17 years old) at Okayama University Hospital.

VGC has several potential toxicities, e.g., it can be carcinogenic and/or teratogenic and can cause azoospermia [6]. Since pharmacists might be at high risk of inhaling VGC when they pulverize VGC tablets, it is important to employ countermeasures to protect them as much as possible from this. Furthermore, pulverizing tablets might decrease the VGC concentrations of the resultant suspensions.

The “simple suspension method” is commonly used to suspend tablets in hot water (55 °C) in Japan because it reduces exposure to toxic medicine powders [7]. However, the use of the simple suspension method to prepare VGC suspensions for nasogastric tube delivery has not been investigated in detail. Furthermore, the stability of the VGC concentrations of such suspensions is unclear.

In the present study, we suspended VGC tablets in hot water using the simple suspension method and examined the stability of VGC, as well as the residual rate of VGC in nasogastric tubes after VGC solution had been passed through them.

## Methods

### Reagents and materials

For the HPLC analysis, a VGC reference standard was purchased from Sigma-Aldrich (MO, USA). Acetonitrile was obtained from Kanto Chemical Co., Inc. (Tokyo, Japan). Sodium dihydrogen phosphate dihydrate and sodium hydrogen phosphate were acquired from Fujifilm Wako Chemicals Ltd. (Osaka, Japan). For the simple suspension method, VGC (450-mg VALIXA® tablets) was provided by Mitsubishi Tanabe Pharma Co. (Osaka, Japan). The gavage syringes (30 mL) and gavage tubes (6 (length: 40 cm), 8 (length: 80 cm), and 12 French (Fr.) (length: 120 cm)) were purchased from JMS Co., Ltd. (Hiroshima, Japan). Twenty-five mM phosphate buffer was created using sodium dihydrogen phosphate dihydrate and sodium hydrogen phosphate.

### Examination of the effects of the simple suspension method on VGC tablets

We prepared VGC solutions under three different conditions. Specifically, an intact VGC tablet (the intact tablet group), a tablet that had been divided in two (the halved tablet group), or a tablet in which the film coating had been cracked (the cracked tablet group) was inserted into a gavage syringe, and then 20 mL of water (25 °C) or hot water (55 °C) was taken up into the gavage syringe containing the tablet. Then, the gavage syringe was left to rest for 10 or 20 min at room temperature (25 °C).

### Stability of VGC after it was suspended in hot water

A VGC tablet whose film coating had been cracked was inserted into a gavage syringe (30 mL). Twenty milliliters of hot water (initial temperature: 55 °C) was taken up by the gavage syringe containing the tablet. Then, the gavage syringe was left to rest for 20, 35, 50, 65, or 80 min under diffuse light conditions at room temperature (500 lx, 25 °C). Furthermore, in order to evaluate the stability of VGC solutions when they are stored for long periods (1, 24, or 48 h), we examined their stability under the following three experimental conditions: under diffuse light storage (500 lx, 25 °C), dark storage (25 °C), and cold dark storage (4 °C) conditions. Next, each solution was diluted 1000-fold using 25 mM phosphate buffer solution. Samples of the resultant solutions were injected into an HPLC system.

### VGC concentrations produced by different metering methods

In order to confirm the VGC concentrations produced by various metering methods, we created three types of VGC solutions. We postulated that half a VGC tablet (225 mg) would usually be prescribed to pediatric patients. Therefore, 1) half a VGC tablet was inserted into a gavage syringe, and then 20 mL of hot water (initial temperature: 55 °C) was taken up into the gavage syringe (the half-tablet group); 2) a VGC tablet was pulverized, half of the pulverized tablet was inserted into a gavage syringe, and then 20 mL of hot water (55 °C) was taken up into the gavage syringe (the half-powder group); or 3) a VGC tablet whose film coating had been cracked was inserted into a gavage syringe, 20 mL of hot water (55 °C) was taken up into the gavage syringe, and then half of the solution (10 mL) was collected (the half-solution group). Then, the gavage syringe was left to rest for 20 min at room temperature (25 °C). The resultant solution was passed through a filter (fine filter F, F162; Forte Grow Medical Co. Ltd., Tochigi, Japan). Then, each solution was diluted 1000-fold using 25 mM phosphate buffer solution. Samples of the resultant solutions were injected into an HPLC system.

### Amounts of VGC remaining in gavage tubes after VGC solution was passed through them

A VGC tablet whose film coating had been cracked was inserted into a gavage syringe. Then, 20 mL of hot water (initial temperature: 55 °C) was taken up by the gavage syringe containing the tablet, before the gavage syringe was left to rest for 20 min at room temperature (25 °C). Then, the solution was passed through a 6, 8, or 12 Fr. gavage tube. Distilled water (20 mL) was then passed through the gavage tube, and a sample of it was injected into an HPLC system.

### Instruments and conditions

The HPLC analysis was carried out with a Shimadzu LC-20A system (Shimadzu Co., Kyoto, Japan). The entire system was controlled using the Shimadzu LC solution software (Shimadzu Co., Kyoto, Japan). A LiChroCART® 125–4.0 Superspher® 100 RP-18(e) (4 μm) column (Kanto Chemical Co., Inc., Tokyo, Japan) was used. Acetonitrile and 25 mM phosphate buffer solution (1:9) was used as the mobile phase at a flow rate of 1.0 mL/min. The column temperature was kept at 40 °C. The UV wavelength used was 256 nm. The analysis time was 20 min. The injection volume was 25 μL. The amount of VGC within the VGC solution at each timepoint was determined using the absolute calibration curve method.

### Preparation of the VGC standard solution for the HPLC

Calibration standards were prepared at five concentrations for the HPLC analysis: 0, 12.5, 25, 50, and 100 μg/mL. The VGC was dissolved in 25 mM phosphate buffer solution.

### Statistical analysis

The data are shown as the mean ± standard deviation (S.D.). The data were analyzed using one-way analysis of variance (ANOVA) followed by Dunnett’s test or Tukey’s test. The significance level was set at *P* < 0.05.

## Results

### Examination of the effects of the simple suspension method on VGC tablets

**Fig. 1:**
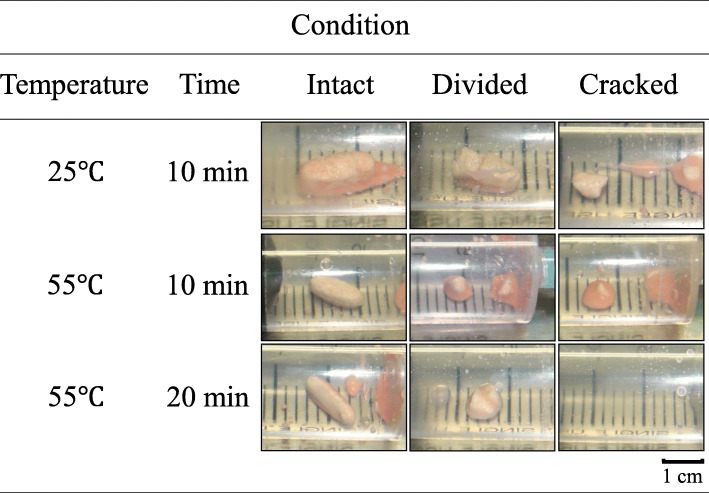
Effects of the simple suspension method on VGC tablets under various conditions

When the tablets were suspended at a water temperature of 25 °C and then left to rest for 10 min, they remained solid in all groups. When the tablets were suspended at a water temperature of 55 °C and then left to rest for 10 min, they also remained solid in all groups. On the other hand, when the tablets were suspended at a water temperature of 55 °C and then left to rest for 20 min, the tablets in the cracked tablet group were completely suspended in the hot water (Fig. 1).

### Stability of VGC after it was suspended in hot water

**Fig. 2:**
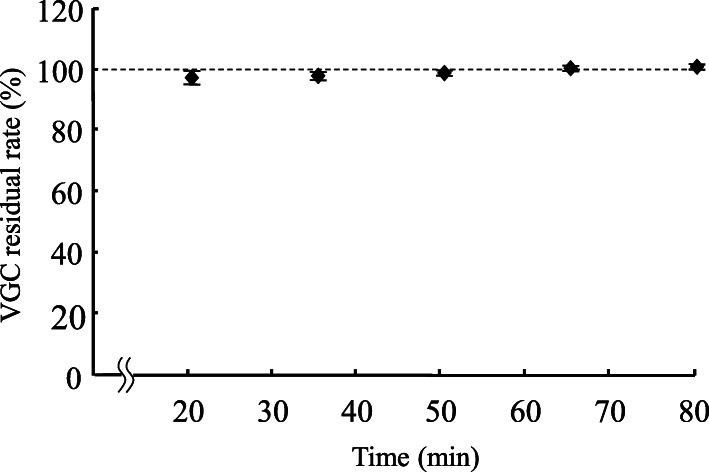
Time-dependent changes in the residual rate of VGC. Each point represents the mean ± S.D. of 6 independent experiments

**Fig. 3:**
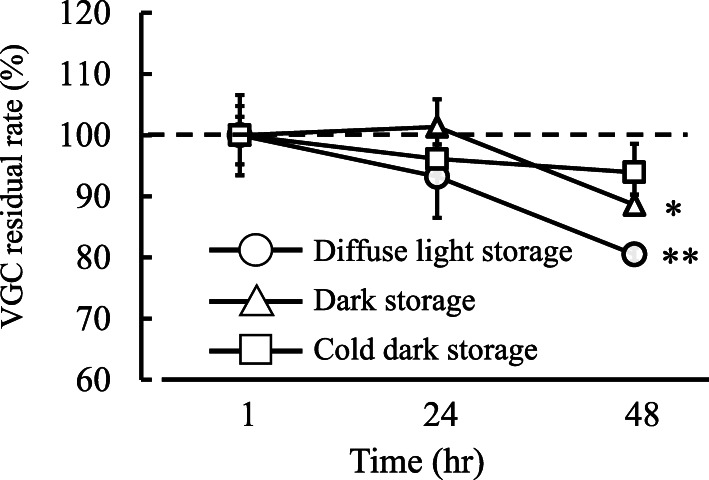
VGC concentrations produced by different storage conditions. Each point represents the mean ± S.D. of 4–5 independent experiments. *: Significant difference between the 1-h and 48-h storage conditions (*P* < 0.05), **: Significant difference between the 1-h and 48-h storage conditions (*P* < 0.01)

Figure 2 shows how the residual rate of VGC changed over time. A residual rate of 100% represented 450 mg of VGC; i.e., the amount of VGC found in one tablet. The residual rate was 99.9 ± 0.9% after 20 min, 99.4 ± 1.6% after 35 min, 99.9 ± 1.3% after 50 min, 101.8 ± 0.3% after 65 min, and 102.4 ± 0.5% after 80 min. Compared with that seen at 20 min, the residual rate of VGC did not change significantly until 80 min. Figure 3 shows the residual rate of VGC from 1 to 48 h under diffuse light storage, dark storage, and cold dark storage conditions. Under the diffuse light storage and dark storage conditions, the residual rate of VGC was significantly decreased at 48 h.

### VGC concentrations produced by different metering methods

**Fig. 4:**
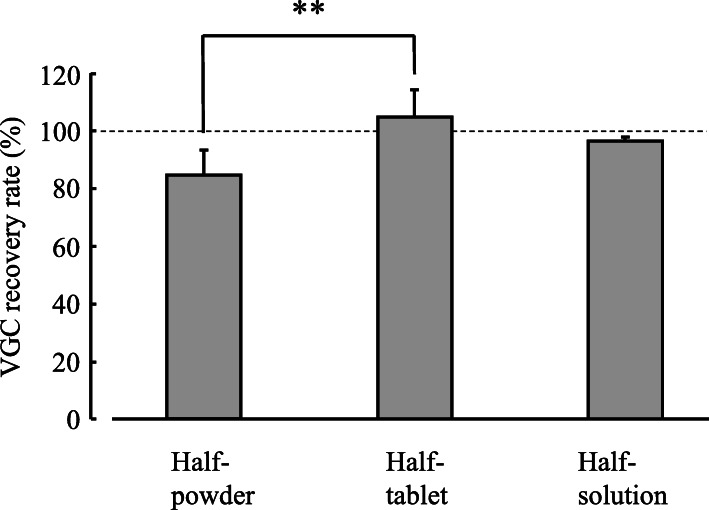
VGC concentrations produced by different metering methods. Each point represents the mean ± S.D. of 6 independent experiments. **: Significant difference between the half-powder group and the half-tablet group (*P* < 0.01)

Figure 4 shows the recovery rates obtained in each group. The VGC concentration was 85.2 ± 8.1% in the half-powder group, 105.0 ± 9.6% in the half-tablet group, and 96.5 ± 1.4% in the half-solution group. The VGC concentration of the half-solution group was not significantly lower than that of the half-powder or half-tablet group. However, the VGC concentration of the half-powder group was significantly lower than that of the half-tablet group [F(2,14) = 7.912, *P* < 0.01].

### Amounts of VGC remaining in gavage tubes after VGC solution was passed through them

**Fig. 5:**
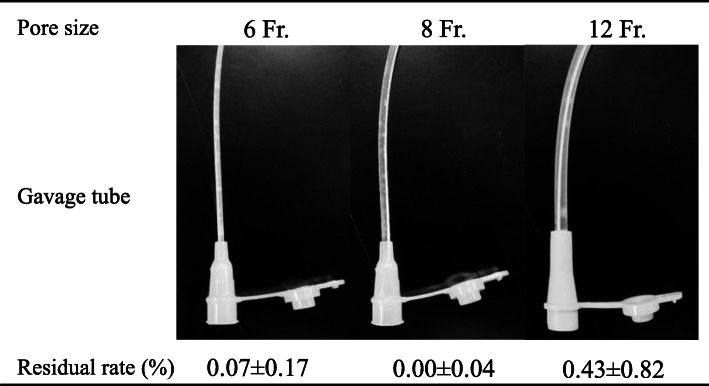
Examination and quantitative determination of residual VGC levels after VGC solution was passed through gavage tubes

Figure 5 shows the residual rates of VGC in gavage tubes of different sizes. The residual rate of VGC was 0.07 ± 0.17% in the 6 Fr. tubes, 0.00 ± 0.04% in the 8 Fr. tubes, and 0.43 ± 0.82% in the 12 Fr. tubes.

## Discussion

In the present study, we demonstrated that suspending VGC tablets whose surface films had been cracked in water with a temperature of 55 °C and then allowing the solution to rest for 20 min was effective at dissolving the tablets. The VGC in the suspensions produced using water with a temperature of 55 °C remained stable for 80 min. In addition, little VGC remained in 6, 8, or 12 Fr. gavage tubes after VGC solution had been passed through them. Moreover, it was suggested that cracking the surface films of VGC tablets is a more effective metering method than preparing powder from VGC tablets. Therefore, we consider that preparations containing the required amount of VGC can be produced using the simple suspension method. It is important to administer the required amount of VGC because it increases patients’ quality of life. Thus, we also consider that the method described in the present study could be useful for maintaining therapeutic effects in patients that are prescribed VGC.

A previous study examined the stability of VGC in aqueous buffer solutions with pH of 3.8 to 11.5 at 37 °C [8]. They found that the half-life of VGC was markedly prolonged in acidic solution. For example, the half-life of VGC was 220 days in pH 3.81 solution, whereas it was 11 h in pH 7.08 solution. Thus, it is necessary to produce an acidic solution when VGC tablets are suspended using the simple suspension method. We previously demonstrated that a suspension of VGC in water produced using the simple suspension method had a pH of 6.64.

It is difficult to apply the simple suspension method to some drugs. In particular, the suspension temperature, dispersibility, and controlled release can all act as limiting factors. Since lansoprazole can be coagulated by hot water, the label information issued by the Food and Drug Administration states that it should be suspended in water at 25 °C. On the other hand, this is not mentioned in the label information given in Japan. While XC (oral anti-tumor drugs: Xeloda® and *cyclophosphamide*) therapy has been developed as a treatment for breast cancer in recent years [9], cyclophosphamide is known to be a carcinogen associated with leukemia and bladder cancer [10]. It decomposes at temperatures of 45 to 53 °C (Japanese Pharmacopoeia), so it cannot be suspended using the simple suspension method. In the present study, we found that VGC tablets whose surface films had been cracked remained stable in suspension for at least 80 min when the suspension was produced using a water temperature of 55 °C.

As a practical test, we examined the delivery of such suspensions by gavage tube and found that the suspensions did not block either the tube or injection syringe. In addition, VGC was hardly detected in gavage tubes after the solution had been passed through them; i.e., the residual rates of VGC in the gavage tubes were extremely low. Indeed, it was reported that the recovery rate of ticagrelor, which is a lipophilic compound similar to VGC, was not affected by the type of tube material (polyurethane, polyvinylchloride, or silicone) used [11]. As VGC is a water-soluble compound, VGC solutions can be administered via gavage tubes. Therefore, we suggest that VGC suspensions created using the simple suspension method can be used for patients that are treated with nasogastric tubes.

Regarding bioavailability, the area under the curve (AUC) value of crushed moxifloxacin tablets in suspension was slightly, but not significantly, lower than that seen after treatment with intact moxifloxacin tablets [12]. However, the AUC of crushed tolvaptan tablets in suspension was significantly lower than that of intact tolvaptan tablets [13]. In the current study, the VGC concentration of the half-solution group was not significantly lower than that of the half-tablet group (it was assumed that half a VGC tablet would usually be prescribed to pediatric patients). In addition, the VGC concentration of the half-solution group was not significantly lower than that of the half-powder group. However, the VGC concentration of the half-powder group was significantly lower (VGC recovery rate: < 90%) than that of the half-tablet group. Therefore, we consider that some of the powder from crushed tablets is lost during the preparation process, which might result in weaker therapeutic effects. Furthermore, under the diffuse light storage and dark storage conditions, the residual rate of VGC was significantly decreased at 48 h. It is recommended that tablets that are dissolved using the simple suspension method should be stored in cold dark conditions.

## Conclusions

Preparing VGC suspensions might protect nursing staff from the adverse effects of aerosolized VGC; reduce the potential loss of the drug during its preparation; and prevent tube-clogging problems, which can arise when insufficiently crushed tablets or a powder that is too thick is administered.

## Data Availability

Not applicable.

## Abbreviations

VGC: Valganciclovir
CMV: Cytomegalovirus
Fr: French
S.D: Standard deviation
ANOVA: Analysis of variance
AUC: Area under the curve
